# Supplementary material for: A Critical View of the Application of the APEX Software (Aqueous Photochemistry of Environmentally-Occurring Xenobiotics) to Predict Photoreaction Kinetics in Surface Freshwaters
Source: Molecules. 2019 Dec 18;25(1):9. doi: 10.3390/molecules25010009 (PMC7017383; doi:10.3390/molecules25010009)
Supplement: Supplementary file 1 [file molecules-25-00009-s001.zip › 3.2.4_gcc-4.4.0.zip!/3.2.4_gcc-4.4.0/3.2.4_gcc-4.4.0/doc/octave/html/interpreter/Special-Functions.html]

Special Functions - Untitled


Next: Coordinate Transformations,
Previous: Utility Functions,
Up: Arithmetic


---

### 17.6 Special Functions

— Loadable Function: [a, ierr] = **airy** (k, z, opt)  
> Compute Airy functions of the first and second kind, and their
> derivatives.
>
> ```
>            K   Function   Scale factor (if 'opt' is supplied)
>           ---  --------   ---------------------------------------
>            0   Ai (Z)     exp ((2/3) * Z * sqrt (Z))
>            1   dAi(Z)/dZ  exp ((2/3) * Z * sqrt (Z))
>            2   Bi (Z)     exp (-abs (real ((2/3) * Z *sqrt (Z))))
>            3   dBi(Z)/dZ  exp (-abs (real ((2/3) * Z *sqrt (Z))))
> ```
>
> The function call `airy (`z`)` is equivalent to
> `airy (0,` z`)`.
>
> The result is the same size as z.
>
> If requested, ierr contains the following status information and
> is the same size as the result.
>
> 0. Normal return.- Input error, return `NaN`.- Overflow, return `Inf`.- Loss of significance by argument reduction results in less than half
>          of machine accuracy.- Complete loss of significance by argument reduction, return `NaN`.- Error—no computation, algorithm termination condition not met,
>              return `NaN`.

— Loadable Function: [j, ierr] = **besselj** (alpha, x, opt)  
— Loadable Function: [y, ierr] = **bessely** (alpha, x, opt)  
— Loadable Function: [i, ierr] = **besseli** (alpha, x, opt)  
— Loadable Function: [k, ierr] = **besselk** (alpha, x, opt)  
— Loadable Function: [h, ierr] = **besselh** (alpha, k, x, opt)  
> Compute Bessel or Hankel functions of various kinds:
>
> `besselj`: Bessel functions of the first kind. If the argument opt is supplied, the result is multiplied by `exp(-abs(imag(x)))`. `bessely`: Bessel functions of the second kind. If the argument opt is supplied, the result is multiplied by `exp(-abs(imag(x)))`. `besseli`: Modified Bessel functions of the first kind. If the argument opt is supplied, the result is multiplied by `exp(-abs(real(x)))`. `besselk`: Modified Bessel functions of the second kind. If the argument opt is supplied, the result is multiplied by `exp(x)`. `besselh`: Compute Hankel functions of the first (k = 1) or second (k = 2) kind. If the argument opt is supplied, the result is multiplied by `exp (-I*`x`)` for k = 1 or `exp (I*`x`)` for k = 2.
>
> If alpha is a scalar, the result is the same size as x.
> If x is a scalar, the result is the same size as alpha.
> If alpha is a row vector and x is a column vector, the
> result is a matrix with `length (`x`)` rows and
> `length (`alpha`)` columns. Otherwise, alpha and
> x must conform and the result will be the same size.
>
> The value of alpha must be real. The value of x may be
> complex.
>
> If requested, ierr contains the following status information
> and is the same size as the result.
>
> 0. Normal return.- Input error, return `NaN`.- Overflow, return `Inf`.- Loss of significance by argument reduction results in less than
>          half of machine accuracy.- Complete loss of significance by argument reduction, return `NaN`.- Error—no computation, algorithm termination condition not met,
>              return `NaN`.

— Mapping Function: **beta** (a, b)  
> For real inputs, return the Beta function,
>
> ```
>           beta (a, b) = gamma (a) * gamma (b) / gamma (a + b).
> ```

— Mapping Function: **betainc** (x, a, b)  
> Return the incomplete Beta function,
>
> ```
>                                                 x
>                                                /
>           betainc (x, a, b) = beta (a, b)^(-1) | t^(a-1) (1-t)^(b-1) dt.
>                                                /
>                                             t=0
> ```
>
> If x has more than one component, both a and b must be
> scalars. If x is a scalar, a and b must be of
> compatible dimensions.

— Mapping Function: **betaln** (a, b)  
> Return the log of the Beta function,
>
> ```
>           betaln (a, b) = gammaln (a) + gammaln (b) - gammaln (a + b)
> ```
>
>
>
>
> **See also:** beta, betainc, gammaln.

— Mapping Function: **bincoeff** (n, k)  
> Return the binomial coefficient of n and k, defined as
>
> ```
>            /   \
>            | n |    n (n-1) (n-2) ... (n-k+1)
>            |   |  = -------------------------
>            | k |               k!
>            \   /
> ```
>
> For example,
>
> ```
>           bincoeff (5, 2)
>                => 10
> ```
>
> In most cases, the `nchoosek` function is faster for small
> scalar integer arguments. It also warns about loss of precision for
> big arguments.
>
>
>
> **See also:** nchoosek.

— Function File: **commutation\_matrix** (m, n)  
> Return the commutation matrix
> K(m,n)
> which is the unique
> m\*n by m\*n
> matrix such that
> K(m,n) \* vec(A) = vec(A')
> for all
> m by n
> matrices
> A.
>
> If only one argument m is given,
> K(m,m)
> is returned.
>
> See Magnus and Neudecker (1988), Matrix differential calculus with
> applications in statistics and econometrics.

— Function File: **duplication\_matrix** (n)  
> Return the duplication matrix
> Dn
> which is the unique
> n^2 by n\*(n+1)/2
> matrix such that
> Dn vech (A) = vec (A)
> for all symmetric
> n by n
> matrices
> A.
>
> See Magnus and Neudecker (1988), Matrix differential calculus with
> applications in statistics and econometrics.

— Mapping Function: **erf** (z)  
> Computes the error function,
>
> ```
>                                    z
>                                   /
>           erf (z) = (2/sqrt (pi)) | e^(-t^2) dt
>                                   /
>                                t=0
> ```
>
>
>
>
> **See also:** erfc, erfinv.

— Mapping Function: **erfc** (z)  
> Computes the complementary error function,
> `1 - erf (`z`)`.
>
>
>
> **See also:** erf, erfinv.

— Mapping Function: **erfinv** (z)  
> Computes the inverse of the error function.
>
>
>
> **See also:** erf, erfc.

— Mapping Function: **gamma** (z)  
> Computes the Gamma function,
>
> ```
>                       infinity
>                       /
>           gamma (z) = | t^(z-1) exp (-t) dt.
>                       /
>                    t=0
> ```
>
>
>
>
> **See also:** gammainc, lgamma.

— Mapping Function: **gammainc** (x, a)  
> Compute the normalized incomplete gamma function,
>
> ```
>                                           x
>                                 1        /
>           gammainc (x, a) = ---------    | exp (-t) t^(a-1) dt
>                             gamma (a)    /
>                                       t=0
> ```
>
> with the limiting value of 1 as x approaches infinity.
> The standard notation is P(a,x), e.g., Abramowitz and Stegun (6.5.1).
>
> If a is scalar, then `gammainc (`x`,` a`)` is returned
> for each element of x and vice versa.
>
> If neither x nor a is scalar, the sizes of x and
> a must agree, and gammainc is applied element-by-element.
>
>
>
> **See also:** gamma, lgamma.

— Function File: l = **legendre** (n, x)  
— Function File: l = **legendre** (n, x, normalization)  
> Compute the Legendre function of degree n and order
> m = 0 ... N. The optional argument, normalization,
> may be one of `"unnorm"`, `"sch"`, or `"norm"`.
> The default is `"unnorm"`. The value of n must be a
> non-negative scalar integer.
>
> If the optional argument normalization is missing or is
> `"unnorm"`, compute the Legendre function of degree n and
> order m and return all values for m = 0 ... n.
> The return value has one dimension more than x.
>
> The Legendre Function of degree n and order m:
>
> ```
>            m        m       2  m/2   d^m
>           P(x) = (-1) * (1-x  )    * ----  P (x)
>            n                         dx^m   n
> ```
>
> with Legendre polynomial of degree n:
>
> ```
>                     1     d^n   2    n
>           P (x) = ------ [----(x - 1)  ]
>            n      2^n n!  dx^n
> ```
>
> `legendre (3, [-1.0, -0.9, -0.8])` returns the matrix:
>
> ```
>            x  |   -1.0   |   -0.9   |  -0.8
>           ------------------------------------
>           m=0 | -1.00000 | -0.47250 | -0.08000
>           m=1 |  0.00000 | -1.99420 | -1.98000
>           m=2 |  0.00000 | -2.56500 | -4.32000
>           m=3 |  0.00000 | -1.24229 | -3.24000
> ```
>
> If the optional argument `normalization` is `"sch"`,
> compute the Schmidt semi-normalized associated Legendre function.
> The Schmidt semi-normalized associated Legendre function is related
> to the unnormalized Legendre functions by the following:
>
> For Legendre functions of degree n and order 0:
>
> ```
>             0       0
>           SP (x) = P (x)
>             n       n
> ```
>
> For Legendre functions of degree n and order m:
>
> ```
>             m       m          m    2(n-m)! 0.5
>           SP (x) = P (x) * (-1)  * [-------]
>             n       n               (n+m)!
> ```
>
> If the optional argument normalization is `"norm"`,
> compute the fully normalized associated Legendre function.
> The fully normalized associated Legendre function is related
> to the unnormalized Legendre functions by the following:
>
> For Legendre functions of degree n and order m
>
> ```
>             m       m          m    (n+0.5)(n-m)! 0.5
>           NP (x) = P (x) * (-1)  * [-------------]
>             n       n                   (n+m)!
> ```

— Mapping Function: **lgamma** (x)  
— Mapping Function: **gammaln** (x)  
> Return the natural logarithm of the gamma function of x.
>
>
>
> **See also:** gamma, gammainc.
